# Glioblastoma Tissue Slice Tandem-Cultures for Quantitative Evaluation of Inhibitory Effects on Invasion and Growth

**DOI:** 10.3390/cancers12092707

**Published:** 2020-09-21

**Authors:** Vasile Sidorcenco, Luisa Krahnen, Marion Schulz, Janina Remy, Donat Kögel, Achim Temme, Ute Krügel, Heike Franke, Achim Aigner

**Affiliations:** 1 Rudolf-Boehm-Institute for Pharmacology and Toxicology, Clinical Pharmacology, Medical Faculty, University of Leipzig, 04109 Leipzig, Germany; v.sidorcenco@gmail.com (V.S.); luisa@gmx.de (L.K.); suffer1@gmx.de (M.S.); 2 Rudolf-Boehm-Institute for Pharmacology and Toxicology, Medical Faculty, University of Leipzig, 04109 Leipzig, Germany; Ute.Kruegel@medizin.uni-leipzig.de (U.K.); heike.franke@medizin.uni-leipzig.de (H.F.); 3 Department of Neurosurgery, Jena University Hospital, 07747 Jena, Germany; 4 Experimental Neurosurgery, Department of Neurosurgery, Neuroscience Center, Goethe University Hospital and German Cancer Consortium (DKTK), Partner Site Frankfurt, 60438 Frankfurt am Main, Germany; JRemy@med.uni-frankfurt.de (J.R.); koegel@em.uni-frankfurt.de (D.K.); 5 Department of Neurosurgery, Section Experimental Neurosurgery and Tumor Immunology, University Hospital Carl Gustav Carus, Technical University Dresden, 01069 Dresden, Germany; Achim.Temme@uniklinikum-dresden.de; 6 German Cancer Consortium (DKTK), Partner Site Dresden, German Cancer Research Center (DKFZ), 69120 Heidelberg, Germany; 7 National Center for Tumor Diseases (NCT), 69120 Heidelberg, Germany

**Keywords:** tissue slice co-cultures, glioblastoma, STAT3, Pim-1, tumor xenografts, ex vivo model, tumor invasion

## Abstract

**Simple Summary:**

Glioblastomas are very malignant and essentially incurable brain tumors. One problem is the extensive penetration of tumor cells into the adjacent normal brain tissue. Thus, the testing of novel drugs requires appropriate tumor models, preferentially avoiding animal studies. This paper describes so-called brain tissue slice tandem-culture systems. They consist of a slice of normal brain tissue and a second layer of tumor tissue. The microscopic analysis of these slice tandem-cultures allows for the simultaneous assessment of single cells invading into the normal brain tissue and the space occupying growth of the total tumor mass. It is shown that the direct application of test drugs onto the slices exerts inhibitory effects on both mechanisms. We thus describe a system mimicking the situation in glioblastoma patients. It reduces animal studies, allows for the direct application of test drugs and the precise quantitation of their inhibitory effects on tumor growth and invasion.

**Abstract:**

Glioblastomas (GBMs) are the most malignant brain tumors and are essentially incurable even after extensive surgery, radiotherapy, and chemotherapy, mainly because of extensive infiltration of tumor cells into the adjacent normal tissue. Thus, the evaluation of novel drugs in malignant glioma treatment requires sophisticated ex vivo models that approach the authentic interplay between tumor and host environment while avoiding extensive in vivo studies in animals. This paper describes the standardized setup of an organotypic brain tissue slice tandem-culture system, comprising of normal brain tissue from adult mice and tumor tissue from human glioblastoma xenografts, and explore its utility for assessing inhibitory effects of test drugs. The microscopic analysis of vertical sections of the slice tandem-cultures allows for the simultaneous assessment of (i) the invasive potential of single cells or cell aggregates and (ii) the space occupying growth of the bulk tumor mass, both contributing to malignant tumor progression. The comparison of tissue slice co-cultures with spheroids vs. tissue slice tandem-cultures using tumor xenograft slices demonstrates advantages of the xenograft tandem approach. The direct and facile application of test drugs is shown to exert inhibitory effects on bulk tumor growth and/or tumor cell invasion, and allows their precise quantitation. In conclusion, we describe a straightforward ex vivo system mimicking the in vivo situation of the tumor mass and the normal brain in GBM patients. It reduces animal studies and allows for the direct and reproducible application of test drugs and the precise quantitation of their effects on the bulk tumor mass and on the tumor’s invasive properties.

## 1. Introduction

Gliomas are the most common primary brain tumors, with glioblastoma (grade IV glioma) being most detrimental and associated with a median survival of only 12–15 months after diagnosis [1,2]. Its particularly poor prognosis that leaves it essentially incurable even after extensive surgery, radio- and chemotherapy is mainly due to the extensive infiltration of tumor cells into the adjacent normal tissue [3]. Thus, while standard treatment strategies mainly focus on the bulk tumor mass, the deeper understanding of tumor cell invasion and the development of novel drugs for its inhibition is of major importance. Classical 2D cell culture or co-culture systems for tumor cell migration, like scratch assays, transwell/Boyden chamber assays or the coating of wells with extracellular matrix (ECM) components like collagen, fibronectin, or laminin [4,5] are rather simple and inexpensive. Yet, such standard methods do not allow for sufficiently assessing the invasive potential due to the absence of matrix components and barrier structures to be encountered by the invading cells. In fact, markedly different protein functions have been previously observed in 2D vs. 3D situation [6,7]. Also, artificial 2D or 3D matrices poorly mimic the in vivo situation, which consists, in addition to the ECM, of multiple cell types that are capable of influencing and interacting with tumor cells, and thus substantially affect their invasive potential [8]. Aiming at systems that provide an organotypic microenvironment with its required cellular complexity [9,10], more recent studies have developed tissue slice models in an air-liquid interface setting [11,12,13,14]. This also included tissue slice co-culture models. Rather than single tumor cells in suspension, which would preferentially grow on the tissue surface, spheroids were implanted onto the brain slice surface or into the brain slice tissue [15,16]. While this approach readily provides some essential features of the host tissue like an authentic ECM, glial-neuronal contact and interaction as well as neuronal connectivity, the spheroids represent only partially the in vivo tumor situation due to the lack of intact tumor tissue structures, which may well affect tumor cell mobility, migration, and invasive potential. On the other hand, in vivo models for a deeper understanding of critical processes underlying cell invasion of human glioblastomas, and in particular for assessing the effects of test substances like specific inhibitors, would have to rely on orthotopic glioblastoma xenografts. Since these models are very costly, time consuming and associated with several ethical and practical issues, the development of meaningful ex vivo systems is urgently needed. Combining the advantages of the intact host microenvironment of organotypic brain tissue slices with an intact tumor tissue, we have developed a glioblastoma tissue slice tandem co-culture setting based on intact tumor tissue derived from mouse xenografts. In particular, this allows for ex vivo studies on the effects of drugs, thus not requiring animals with the exception of the tissue donors and thus representing an alternative to the use of animals for scientific purposes. While in this study, tumor xenograft material was used (with a very small number of animals required since the tumor tissues are then propagated into many ex vivo samples), this may be even extended toward primary tumors from patients.

## 2. Results

### 2.1. Tissue Slice Preparation and Cultivation

**Figure 1:**
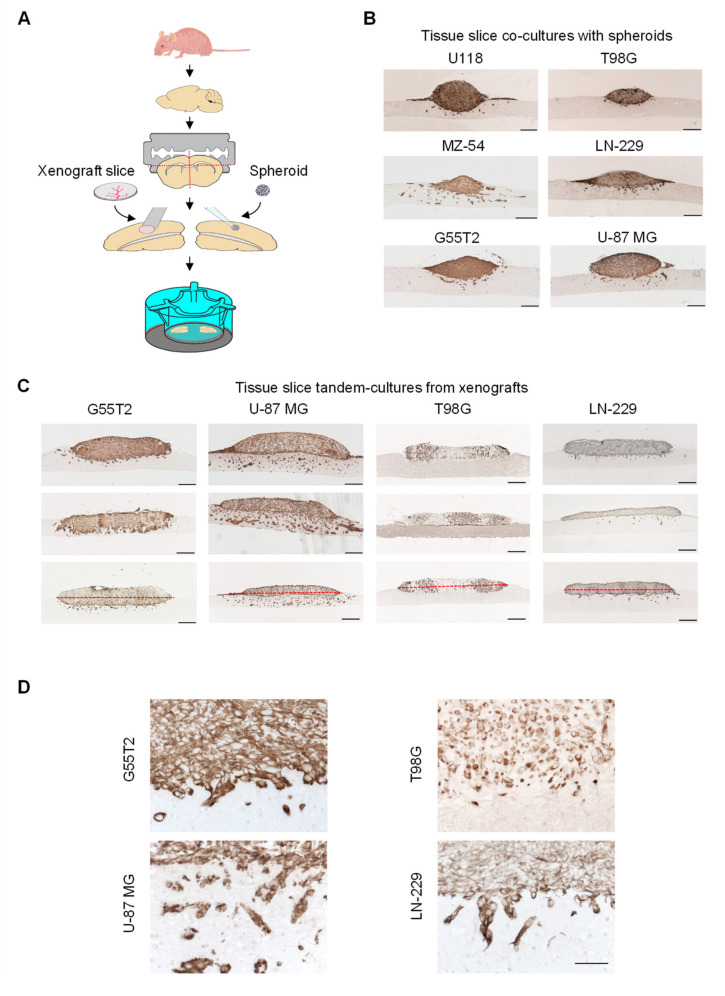
(**A**) Scheme of tissue slice tandem-culture preparation and cultivation, relying on the co-culture of a xenograft tissue slice on top of a normal brain tissue slice and thus replacing spheroid co-culture models based on established cell cultures. (**B**) Microscopic pictures of spheroid co-cultures and (**C**) xenograft tissue slice tandem-cultures (in triplicates) stained for Vimentin, based on different cell lines. Images were taken from tissue stained after one week of cultivation. (**D**) Higher magnification of representative segments from Figure 1C. Scale bars: 200 µm (**B**,**C**), 50 µm (**D**).

Tissue slice tandem co-cultures were set up as depicted in Figure 1A. In the following, the term “spheroids” always refers to established cell cultures while the term “xenografts” indicates the use of tumor explants. Prior to preparing the 300 µm tissue slices, the normal mouse brain was embedded into agarose as a “carrier matrix” in order to allow more even cutting of the tissue. Cortical brain segments were selected as the basal layer underneath glioblastoma xenograft tissue slices or spheroids. While cultured spheroids were already comparable in size, glioblastoma xenograft tissue slices were processed into equal sizes by excising circular segments of 2 mm in diameter from peripheral, non-necrotic tumor areas using a biopsy punch.

This also allowed for obtaining multiple samples from one tumor, thus substantially reducing required animal numbers. Both, spheroids and punched tissue xenograft segments attached well to the underlying normal brain tissue and remained viable over the whole period of the experiments, i.e., for at least seven days. Immunohistochemical staining of paraffin sections for Vimentin allowed for accurately distinguishing between tumor cells (positive) and cells of the neuronal and glial host tissue (negative). From higher magnifications (see below) and the observed staining patterns, the co-staining of blood vessels could be excluded. In case of a visible gap between the tumor and the normal tissue, slides were discarded.

### 2.2. Characterization of Tumor Growth and Invasion/Penetration

**Figure 2:**
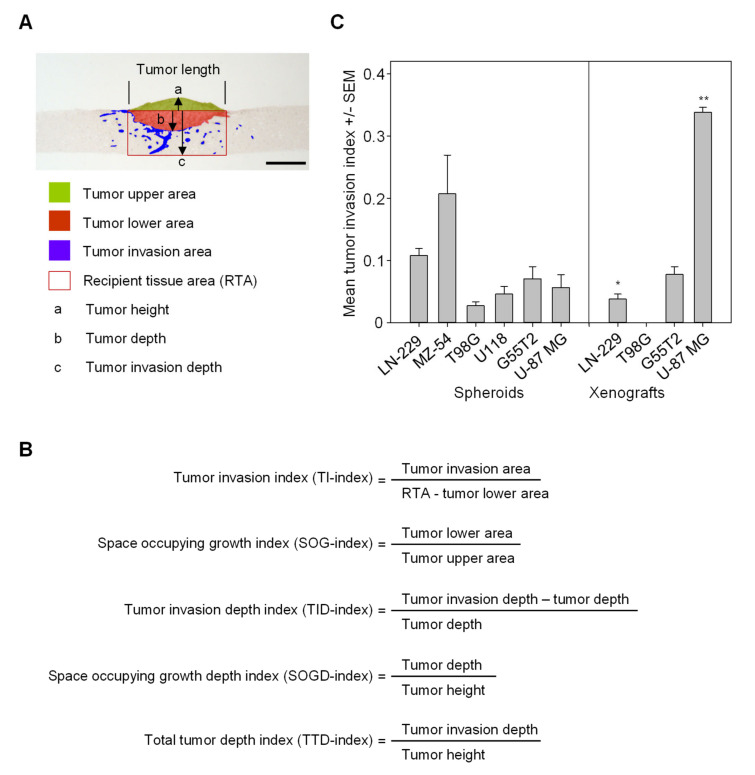
(**A**) Definition of areas for quantitative distinction between tumor growth, space occupying tumor growth (tumor mass effect), and tumor cell invasion. Other parameters (tumor height, tumor depth, tumor invasion depth and recipient tissue area) are shown as well. (**B**) Definition of “tumor invasion index” and “space occupying growth index,” for exact quantitation. Additionally, indices related to tumor depth and tumor invasion depth are defined. (**C**) Tumor invasion index values of spheroid co-cultures and xenograft tandem-cultures, based on different cell lines. Asterisks indicate statistically significant (* = *p* < 0.05, ** = *p* < 0.01) differences of xenograft tandem-cultures vs. their spheroid co-culture counterparts.

When analyzing spheroid-based co-cultures at day 7, major differences between different cell lines were observed with regard to overall spheroid growth, space occupying growth of the bulk tumor mass into the normal brain (slice) without dissemination of individual invading cells (“space occupying tumor growth”) and invasion of single cells upon leaving the bulk tumor/spheroid mass (“tumor invasion”; Figure 2). More specifically, while a somewhat comparable space occupying tumor growth was observed in U118, MZ-54, and G55T2 cell spheroids, substantial cell invasion was only observed in the case of MZ-54 spheroids and to a lesser extent in their U118 or T98G counterparts (Figure 1B). For better clarity, in the same images red dotted lines were included indicating the lower edge of the normal brain tissue layer and the “horizon,” i.e., the border between the tumor upper and lower area (Figure S1A,D). Additionally, co-staining of sequential sections for GFAP or S-100 confirmed the distinction between tumor and underlying normal brain tissue. For additional clarity, borders between the tumor and the underlying normal brain tissue were marked by a thin red line in the Vimentin-stained pictures (Figure S1B, upper panels), and the exactly same line was copy-pasted and used in the corresponding IHC stains of the normal brain tissue (Figure S1B, lower panels; IHC with tumor tissue remaining unstained). These complementary staining patterns allowed for the specific distinction between the tumor vs. the underling normal brain tissue. More importantly, the higher magnification in Figure S1C, which again uses identical red lines for delineating the border between tumor and underlying normal brain tissue, showed infiltrating cells (Figure S1C, left, on the right side of the picture). These were found to be clearly located in the normal brain tissue, as determined by the corresponding immunohistochemistry (IHC) for S-100 (Figure S1C).

Based on this analysis, spheroids derived from LN-229, U118, or G55T2 cells showed an intermediate profile, with space occupying properties as well as some single cell invasion. U-87 MG cell spheroids exhibited very little invasion into the normal brain and grew mainly above the host tissue. Given the fact that cell invasion into the surrounding tissue is a major hallmark of glioblastoma, this poses a major limitation with regard to this model resembling the in vivo situation. 

**Figure 3:**
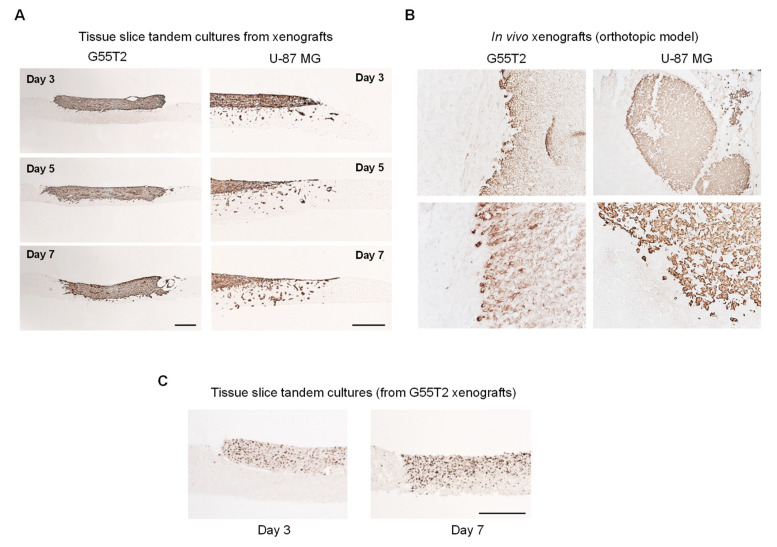
(**A**) Vimentin staining of time-dependent tumor growth and invasion in xenograft tandem-cultures, based on G55T2 (left) or U-87 MG cells (right). (**B**) Orthotopic xenografts based on G55T2 (left) or U-87 MG cells (right) injected into the mouse striatum, stained for Vimentin. (**C**) Proliferating cells in G55T2 xenograft tandem-cultures, as determined by Ki-67 staining. Scale bars: 200 µm.

In contrast, more prominent differences were observed when switching from spheroids obtained in cell culture to tumor xenograft punches for tissue slice tandem co-culture (Figure 1C, Figure S1D). Xenografts from four cell lines (G55T2, U-87 MG, T98G, and LN-229) were selected and directly compared in the same setting. This selection was mainly based on the availability of xenograft tissue material, i.e., on sufficient tumorigenicity of the respective cell line. In tandem-cultures containing G55T2 xenografts, a profound space occupying growth behavior was observed, in some cases leading to complete substitution of the normal brain layer after seven days (Figure 1C, Figure S1D, and Figure 3A). Interestingly, space occupying growth was also found to be dependent on the host tissue since it was more prominent in tandem-cultures prepared from cortex than from striatum (Figure S2). In this paper, studies were restricted to cortex; however, this does not exclude using white matter as underlayer which will be of additional interest from a clinical viewpoint.

In contrast to G55T2 cell xenografts, tissue slice tandem-cultures based on U-87 MG xenografts revealed profound single cell invasion. On the other hand, space occupying growth properties were largely absent and the bulk tumor grew rather to the outside and on the surface of the normal brain layer. T98G xenografts showed some space occupying growth but were devoid of any single cell invasion, while their LN-229 cell counterparts revealed little space occupying growth but some invading cells (Figure 1C). These differences in cell invasion properties were even more obvious at higher magnification, which also allowed us to differentiate between single invaded cells and multi-cell clusters, and confirmed the absence of blood vessel staining (Figure 1D and Figure S3). Thus, this panel revealed the whole spectrum of possible growth characteristics with regard to space occupying growth and single cell invasion.

### 2.3. Quantitation of Space Occupying Tumor Growth and Tumor Cell Invasion

In order to quantitatively differentiate between space occupying growth of the bulk tumor and tumor cell invasion, Vimentin-stained sections were scanned and analyzed using ImageJ as described in the Materials and Methods. Areas were defined as shown in Figure 2A for distinguishing between (i) the tumor upper area, representing the (growing) initial tumor mass, (ii) the tumor lower area, i.e., the space occupying tumor growth, and (iii) the amount of cells having invaded into the normal brain tissue, defined as the “tumor invasion area.”

Tumor height, tumor depth, and tumor invasion depth (the latter two referring to the tumor lower area and the tumor invasion area, respectively) were also defined (Figure 2A). Since absolute values of the tumor invasion area will depend on the overall size of the tumor piece, the rectangular area underneath the tumor was determined as well (recipient tissue area; RTA). Based on these data, values of several indices could be calculated (Figure 2A). The tumor invasion index (TI-index) was defined as the ratio between tumor invasion area and the RTA. Additionally, the space occupying growth index (SOG-index) was defined as a second parameter, quantitatively describing the space occupying growth of the bulk tumor mass without individual tumor cell invasion (ratio between tumor lower area and tumor upper area; Figure 2A). Other index values referring to the tumor depth, tumor height and tumor invasion depth; i.e., the maximum distances from the ‘horizon’ (a, b and c in Figure 2A), were defined as tumor invasion depth index (TID-index), space occupying growth depth index (SOGD-index), and total tumor depth index (TTD-index; Figure 2B).

When applying these parameters to the spheroid co-cultures from Figure 1B and the tissue slice tandem-cultures (Figure 1C) for calculating the tumor invasion index, the observed differences were accurately quantitated (Figure 2C). Notably, in the case of the U-87 MG xenograft tandem-cultures a profound and statistically significant ~6-fold increase in cell invasion as compared to the corresponding spheroid co-cultures was observed (Figure 2C, right), thus better mimicking the highly invasive potential of glioblastoma. In other cases, no (G55T2) or minor (LN-229) differences with slightly decreased tumor invasion were observed. It should be kept in mind, however, that the tissue slice tandem-cultures represent more closely the in vivo situation, with tumor cells having to escape from an intact tissue environment rather than from more artificial spheroids. This was particularly obvious in the case of T98G xenografts where no single cell invasion was observed.

According to the definition given in Figure 2A, the space occupying growth index was determined as well. In the xenografts of three out of four cell lines (T98G, G55T2, and U-87 MG), some minor reductions in the growth potential were observed as compared to their spheroid counterparts, again indicating altered growth characteristics of intact tumor tissue (Figure S4A). This effect was less obvious from the total tumor depth index (TTD-index; Figure S4B), while the space occupying growth depth index (SOGD-index) revealed even more profound tumor growth into the host tissue in the case of the G55T2 xenografts compared to spheroids (Figure S4C).

### 2.4. Further Characterization of Tissue Slice Xenograft Tandem-Culture Properties in Comparison to Orthotopic Xenografts

The time-dependent assessment of tumor growth revealed an almost complete penetration of G55T2 tumor xenografts already at day 5, with the tumor mass reaching the bottom of the normal brain slice at day 7 (Figure 3A, left; Figure S5A, left; Figure S2, left). This was largely in line with growth properties of orthotopic G55T2 xenografts in the striatum, which showed a space occupying tumor mass with fairly well-defined edges (Figure 3B, left). Ex vivo tumor growth in tissue slice xenograft tandem-culture was accompanied by substantial cell proliferation, as determined by Ki-67 staining, also indicating the preservation of high cell viability over the whole time period (Figure 3C; Figure S5B). Strikingly, in the case of U-87 MG xenografts, cell invasion started very early, with some cells reaching the bottom of the host tissue slice already at day 3 (Figure 3A, right; Figure S5A, right). These invasive properties were more profound compared to the in vivo situation where orthotopic xenografts showed rather well-defined edges, with only a few cells starting to disseminate into the host tissue (Figure 3B, right).

### 2.5. Validation and Testing of the Quantitation of Tumor Invasion Index and Space Occupying Growth Index

For further validation of our quantitative analysis method (determination of the tumor invasion index and the space occupying growth index), tissue slice co-cultures with spheroids from MZ-54 wildtype vs. MZ-54 STAT3 knockout (KO) cells were compared (Figure S6A,B). Signal transducer and activator of transcription 3 (STAT3) has been shown previously to be a key signaling protein driving major hallmarks of cancer, including proliferation and cell survival, and to represent a promising target for the development of targeted glioblastoma therapies. It is frequently overexpressed and overactivated in GBM and associated with the most aggressive and treatment-resistant mesenchymal subtype [17,18].

Vimentin staining for glioblastoma cells revealed an overall decreased invasion upon STAT3 knockout, as to be expected from our previous findings [19]. This was confirmed by a ~50% reduction of the tumor invasion index in the STAT3 depleted cells (Figure S6B). Switching to a situation closer to therapy and drug assessment, wildtype spheroid/tissue slice tandem-cultures were treated with a combination of a STAT3 inhibitor (Stattic) and the Pim1 inhibitor (SGI-1776). The proto-oncogenic survival kinase Pim1 is overexpressed in glioblastoma and has been associated with tumor cell survival, proliferation and anti-apoptotic effects [20,21]. Again, a marked reduction of the tumor invasion index was observed (Figure S6C), indicated by a ~50% decreased tumor invasion index (Figure S6D, left). In parallel, the space occupying tumor growth was markedly reduced as well, as determined from the space occupying growth index (Figure S6D, right). Thus, our method accurately quantitates tumor growth and invasion properties upon treatment, and it was subsequently used to monitor effects in our xenograft tissue slice tandem-cultures.

### 2.6. Pim1 Inhibitor Therapy Studies in Tissue Slice Tandem-Cultures

**Figure 4:**
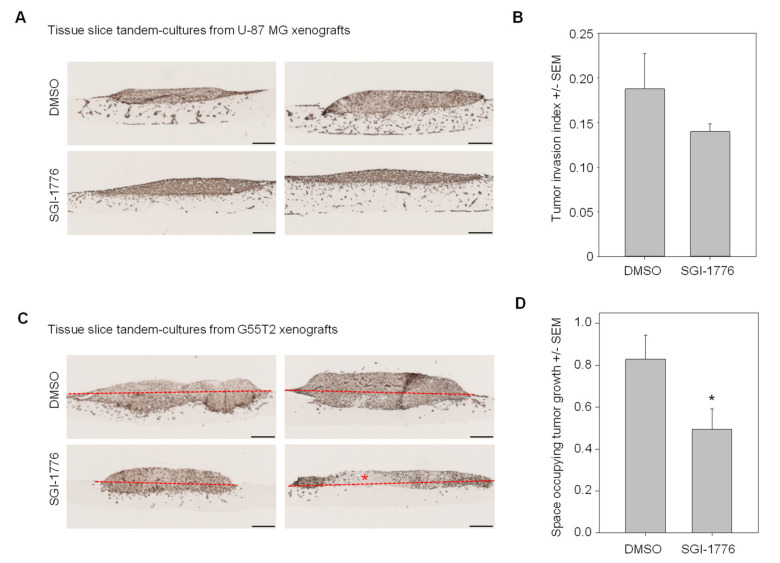
Treatment of xenograft tandem-cultures based on (**A**,**B**) U-87 MG or (**C**,**D**) G55T2 cell xenografts with the Pim1 inhibitor SGI-1776 vs. solvent control. (**A**,**C**) representative microscopic pictures of Vimentin staining (asterisk: necrotic area). (**B**,**D**) Results of the quantitation of the tumor invasion index or space occupying tumor growth. Scale bars: 200 µm; * = *p* < 0.05.

Since their profound invasive potential was the hallmark of U-87 MG xenografts growing in tissue slice tandem-cultures, we assessed Pim1 inhibitor effects on the tumor invasion index. Upon treatment with SGI-1776 applied at a subtoxic concentration (5 µM), fewer cells were found to invade into the normal brain host tissue or to even reach the lower side of the tissue slice (Figure 4A; Figure S7A). This was paralleled by a decrease in the tumor invasion index upon inhibitor treatment (Figure 4B). In contrast, in G55T2 xenograft tissue slice tandem-cultures allowed for monitoring differences in the space occupying growth (Figure 4C and Figure S7B). This was found reduced by ~40% upon inhibitor treatment (Figure 4D).

## 3. Discussion

Beyond the mere growth of a primary tumor, other processes are major contributors to the malignancy, limitations in treatment options and thus the prognosis of cancer. Especially in glioblastomas, this involves aggressive tumor cell invasion, requiring authentic and reproducible models for studying the underlying tumor biological mechanisms and assessing novel drugs for specific interference with this major therapeutic obstacle. Considering the 3R principle and the complexity of appropriate in vivo models, this calls for powerful ex vivo models. 

Our data demonstrate that (i) tissue slice tandem cultures are well-suited for studying local glioblastoma growth and invasion, and (ii) a setting based on tumor xenografts shows advantages over spheroids for analyzing the invasive and space occupying potential of glioblastomas. As compared to in vivo studies, it only requires a very limited amount of tumor material obtained from a small number of animals. Despite being an ex vivo model, it offers (iii) all advantages regarding intact tumor material, allowing a clear distinction between tumor cell invasion and space-consuming tumor growth, and (iv) thus allows for the accurate quantitation of inhibitory effects upon direct application of drugs, based on (v) a straightforward method of scanning and analyzing immunohistochemical sections. Our data also indicate that s.c. xenografts are sufficient to fulfill these requirements, abolishing the need to use considerably more problematic orthotopic glioblastoma xenografts, and that a large number of samples can be obtained from just one xenograft. Thus, this approach clearly addresses the 3R principle with regard to markedly reducing animal numbers and refining the model systems by using an in many ways better-suited ex vivo setting. In this regard, it is tempting to speculate that, in the light of current developments, mixed spheroid cultures based on the co-culture of defined combinations of tumor (here: glioblastoma) cells with other stroma and immune cells may eventually lead to settings that are able to replace xenograft tandem cultures, thus eliminating the need for tumor-bearing animals. The same is true with regard to the possibility of using primary tumor material from patients, only limited by the availability of sufficient amounts of tumor mass. However, our approach of using a biopsy punch on tumor slices described here also provides an efficient tool of obtaining as many samples as possible, with very comparable dimensions. Thus, the analysis of primary brain explants which may differ from xenografts with regard to proliferation rates and brain infiltration will be of particular interest. While it is feasible to use dissociated primary brain tumor cells for spheroid co-cultures, the intact tumor tissue can be used for the tandem cultures described here. The cultivation in an air-liquid interface (ALI) setting is of major importance in all experiments employing intact tumor material. Initially, our major concern was the possible malnutrition of the tumor tissue, being sub-optimally supplied with nutrients from the medium through the layer of normal brain. However, from our results this can be excluded. Likewise, our data do not support the occurrence of disrupted tissue structure of the normal brain tissue underneath the tumor.

Of note, the model established in this study proved to be very powerful in monitoring effects upon therapeutic intervention. STAT3 and its inhibition has been explored rather extensively with regard to its oncogenic role and the identification of novel therapeutic targets in glioblastoma [19,22,23]. In line with this, tumor inhibitory effects are observed in our model as well. Beyond small molecule inhibitors, these approaches of STAT3 inhibition may well be extended towards other strategies like gene knockdown, as shown recently in a preclinical study in this pathology [24]. The role of the pro-survival kinase Pim1 and its inhibition are less established but have been explored as well [20,21,25,26]. Interestingly, while both inhibitors employed here have been shown to exert tumor-inhibitory effects, this study and our model also demonstrate that the major underlying effects may be different (inhibition of bulk tumor growth vs. singe cell invasion). This also suggests an additional benefit of combination therapies, by parallel acting on different tumor-biological processes, which can be accurately analyzed in this setting. Still, in the age of cancer immunotherapy, limitations of this model are obvious with regard to the absence of an immunocompetent setting. Again, it will be interesting to further extend our model toward mixed spheroids that include immune cells grown on an immunocompetent brain setting or by using xenograft material from humanized mice.

Another major issue in several models is the definition of objective and quantitative readouts. While space-assuming growth and tumor cell infiltration can be quantitated better in this ex vivo model as compared to the in vivo situation, it still relies on rather tedious microscopic evaluation, impairing high-throughput analyses of various drugs or drug combinations. Other readouts, perhaps based on non-microscopic measurements, are thus needed. Indeed, initial data from our lab on chemotherapy-mediated alterations in cell cycle-genes as determined from tissue slice lysates indicate that at least in some cases these approaches will be feasible. While these lysate-based approaches would not address the microscopic quantitation of invasion and space occupying growth as the major advantage described here, both methods may well complement each other, thus providing even more information hardly obtainable from an in vivo experiment. Studies on other established or putative drivers of tumor cell invasion will further clarify the potential of tandem slice cultures, for drug assessment as well as for obtaining further insight into the tumor biology of invasion. Beyond tumor cells, this will also involve the role of the surrounding tissue; i.e., the role of other non-tumor cells in the tumor as well as the question which normal tissue to be used as the bottom layer. This will thus address in further detail, and with ex vivo precision, the role of the tumor environment. Taken together, this ex vivo systems not only limits the necessary number of animals to almost zero, but is also better suited than in vivo experiments when it comes to precisely highlighting biological details, for example regarding the role of the exact location of the glioblastoma.

## 4. Materials and Methods

### 4.1. Animals

Athymic nude mice (Crl: CD1-Foxn1nu, Charles River Laboratories, Sulzfeld, Germany) and NOD/SCID/IL2r gamma (null) mice (Jackson Laboratory, Bar Harbor, ME, USA) were housed under standard conditions, representing contemporary best practice, and allowed access to lab food and water ad libitum. All individuals involved with the care and use of animals were trained and skilled to the high levels of competency required by the authorities. Since this study explicitly aimed at minimizing numbers of animals by establishing and using ex vivo methods for experimentation, only 17 mice were needed for the whole study.

All mouse experiments were performed strictly according to the national regulations of animal welfare and ethically reviewed/approved by the local committee on animal welfare and the local authorities (Landesdirektion Sachsen, Germany). This included measures to reduce suffering and pain management (where applicable), the definition of humane end points (maximum tumor xenograft sizes or other end points; not reached in this study) and euthanasia methods, the precise definition and weighing of likely or possible adverse effects. The design and execution of the animal experiments was under strict and careful consideration of the 3R principles. Experimental methods and analysis of results followed established international standards, in particular fully complying with the ARRIVE guidelines.

### 4.2. Cell Culture and Spheroid Generation

Glioblastoma cell lines LN-229, U-87 MG, U118 and T98G were obtained from the American type culture collection (ATCC). The cell line G55T2 was kindly provided by Dr. Katrin Lamszus [27], and the cell line MZ-54 has been described previously [28]. For establishment of the MZ-54 STAT3 knockout (KO) cell line, the CRISPR/Cas9 method [29] with Cas9 derived from *Streptococcus pyogenes* was used. For this purpose, two plasmids containing different small single-guide RNAs (design Benchling, Eurofins Genomics) ligated into the pSpCas9(BB)-2A-Puro vector (Addgene) were transfected in parallel into MZ-54 cells, using Lipofectamine 3000 (Thermo Fisher Scientific, Waltham, MA, USA) according to the manufacturer’s instructions. Cells were selected from day 2 to day 6 post-transfection with 1 µg/mL Puromycin and afterwards isolated for establishing stable single cell clones. KO clones were identified by genotyping PCR, sequencing via Microsynth Seqlab and Western Blot analysis.

All cell lines were cultivated in a humidified atmosphere under standard conditions (37 °C, 5% CO_2_) in IMDM (Iscove’s Modified Dulbecco’s Media; Sigma-Aldrich, Steinheim, Germany), supplemented with 10% fetal calf serum (FCS) and 2 mM stable L-alanyl-L-glutamine (Biochrom, Berlin, Germany), and frequently tested for absence of mycoplasma contamination. For spheroid formation, 2.5 × 10^4^ cells in 200 µL cell culture medium were seeded into ultra-low attachment U-shaped 96-well plates (Brand Scientific, Wertheim, Germany) and grown for five days.

### 4.3. Tumor Cell Xenografting and Tissue Slice Preparation

To generate tumor xenografts, 5 × 10^6^ cells suspended in 150 µL phosphate-buffered saline (PBS) pH 7.4 (Sigma-Aldrich) were subcutaneously injected into both flanks of ~8 weeks old mice (U-87 MG, G55T2: Crl: CD1-Foxn1nu; LN-229, T98G: NOD/SCID/IL2r gamma (null) mice). The xenografts were allowed to grow until the desired size of ~10 mm in diameter was reached. The generation of orthotopic brain xenografts in Crl:CD1-Foxn1nu mice was performed essentially as described previously [30]. For this, 1 × 10^6^ U-87 MG cells or 3 × 10^5^ G55T2 cells were injected. After ~3 weeks, brains were explanted, cryo-sectioned or paraffin-embedded, and further processed as described below.

Cortical brain tissue slices were prepared from ~4-month-old mice and cultivated as previously described [31]. Briefly, the brain was carefully removed under aseptic conditions immediately after sacrificing the mice and placed into a petri dish containing warm 1.5% agarose solution. After solidification on ice, a block containing the cortex or the striatum was prepared and glued onto the vibratome specimen holder using Histoacryl (Braun, Melsungen, Germany). Coronal sections were cut at cortical levels using a Leica VT1200S Vibratome (Leica Microsystems, Nussloch, Germany). In the vibratome buffer tray, the specimen holder was submerged in ice-cold preparation medium (Minimal essential medium (Thermo Fisher Scientific), 2 mM glutamine (Gibco/Thermo Fisher Scientific), 50 µg/mL Gentamicin (VWR International, LLC, Solon, OH), pH 7.3. The vibratome was programmed to cut slices at a thickness of 300 µm at a speed of 0.2 mm/sec. The preparation of 300 µm-thick xenograft tumor tissue slices was performed in a similar way. After explantation, the xenografts were temporarily stored in ice-cold PBS prior to embedding in a 4% agarose mold and slices were cut as above. For obtaining smaller pieces of identical size, a skin biopsy punch (KAI Europe, Solingen, Germany) was used for excising circular segments of 2 mm in diameter from peripheral tumor areas which were determined as non-necrotic by macroscopic and stereo-microscopic inspection.

### 4.4. Tissue Slice Co-Culture Cultivation and Treatment

All subsequent steps were performed under a sterile working bench equipped with a stereomicroscope. Buffers and procedures were according our protocols used for studies on axon regeneration/neuronal outgrowth in organotypic slice co-cultures under ex vivo conditions [31]. Tissue culture (TC) 6-well plates (Sarstedt, Nümbrecht, Germany) were used in conjunction with TC inserts (Sarstedt) with a semi-permeable porous membrane, for holding the tissue slices at the air-liquid interface. Then, 1 mL incubation medium (50% Minimal essential medium, 25% Hank’s Balanced Salt Solution (Gibco), 25% heat-inactivated horse serum (Sigma-Aldrich), 2 mM glutamine, 0.625% glucose; pH 7.2) containing 50 µg/mL Gentamicin was added to each well of the TC plate. After that, the inserts were introduced to the well, for warming up and equilibration of the inserts and the medium in an incubator at 37 °C, 5% CO_2_ for at least 20 min. The murine brain tissue slices were transferred from the ice-cold preparation medium onto the membranes. Tumor spheroids were picked from the culture well using a 10 µL pipette and carefully transferred onto the cortical area of the brain tissue slice. Similarly, tumor xenograft pieces were transferred from the petri dish with ice-cold PBS onto the brain tissue slices. The TC 6-well plates containing the tissue slice tandem-cultures were incubated under standard cell culture conditions for seven days, unless stated otherwise. The medium was changed after 24 h and then every other day. For inhibitor treatment, the STAT3 inhibitor Stattic and/or the PIM-1 kinase inhibitor SGI-1776 were added at 5 µM final concentration to the incubation medium directly after the tissue slice tandem co-culture preparation, with the corresponding volumes of solvent (DMSO) serving as negative control. Subsequently, the medium was changed to new inhibitor-containing medium every other day as above, with additional 25 µL being directly pipetted onto the tissue slices for direct application. 

### 4.5. Immunohistochemical Analysis

Upon harvesting (after seven days of cultivation, unless indicated otherwise), tissue slice tandem-cultures were fixed for 2 h in a solution containing 4% paraformaldehyde (Merck, Darmstadt, Germany), 0.1% glutaraldehyde (Serva, Heidelberg, Germany), and 0.2% picric acid (Sigma-Aldrich) in 100 mM Sorensen’s Phosphate Buffer (SPB, pH 7.35), then washed thoroughly and stored at 4 °C. To physically protect the fragile tissue slice tandem-cultures in the subsequent steps, they were pre-embedded in a warm 2% agarose/2.5% gelatine mix in water. Upon cooling, this provided a protective porous matrix around the specimen, sufficiently permeable for organic solvents and sufficiently flexible for preventing osmotic distortion [32]. For complete solidification, blocks containing the embedded tissue were subsequently stored in 70% ethanol for 72 h. Dehydration in a series of graded ethanol was performed in an automated tissue processor (Citadel 1000, Shandon/Thermo Fisher Scientific, UK), prior to paraffin embedding (Tissue-Tek Embedding Console Model 4715, Sakura, Japan). From paraffin blocks, 5 µm sections were prepared in a Leica Reichert-Jung Biocut 2035 microtome (Leica Biosystems, Nussloch, Germany), mounted onto polylysine histological glass slides (Gerhard Menzel, Braunschweig, Germany) and left to stretch and dry on a Leica HISTOPLATE stretching table (Leica Biosystems) heated to 43 °C.

For de-paraffination, slides were incubated 2 × 5 min in Neo-Clear (Merck, Darmstadt, Germany), followed by a series of decreasing ethanol concentrations (100%, 96%, 70%) and a final short washing step in distilled water. Antigen retrieval was done by microwave pre-treatment in heat-induced epitope retrieval (HIER; [33]) buffer solutions (1 mM EDTA-HIER-buffer, pH 8.0 for anti-Ki67 or 10 mM sodium citrate-HIER-buffer, pH 6.0 for anti-Vimentin, anti-GFAP and anti-S-100, respectively), by boiling for 20 min in a microwave. After cooling at room temperature for 20 min, slides were washed 2 × 5 min with SPB at room temperature. For blocking of nonspecific binding sites, slides were treated with blocking solution (SPB containing 0.3% Triton X-100, 1% bovine serum albumin, 5% normal horse serum) at room temperature for 30 min, followed by incubation with the primary antibodies (mouse anti-Ki67 (1:100), mouse anti-Vimentin (1:1000), rabbit anti-GFAP (1:250) or rabbit anti-S-100 (1:200); all from DakoCytomation, Glostrup, Denmark) in the blocking solution at 4 °C overnight. After washing 2 × 5 min in SPB, slides were treated for 15 min with 3% H_2_O_2_ diluted in SPB, to block endogenous peroxidase activity. After intensive washing in SPB, slides were incubated with a biotinylated anti-mouse or anti-rabbit secondary antibody (immunoglobulin IgG (H + L); 1:350; Vector Laboratories, Burlingame, CA) in blocking solution for 50 min at room temperature. For detection, the streptavidin/biotin technique was performed using StreptABComplex/HRP (DAKO Denmark, Glostrup, Denmark; 50 min incubation time). For visualization, the sections were incubated in 1% 3,3′-diaminobenzidine tetrahydrochloride (DAB; Sigma-Aldrich)/0.3% H_2_O_2_ in SPB (pH 7.2) until the brown staining was observed under the microscope. After washing, the stained sections were dehydrated in a series of graded ethanol, processed through n-butylacetate (Carl Roth, Karlsruhe, Germany), coverslipped with Entellan (Merck) and left to dry overnight. Control experiments were carried out without the primary antibody.

Microscopic pictures were taken with a Zeiss Axioskop upright microscope, equipped with a Zeiss AxioCam ICc 1 camera (Carl Zeiss, Jena, Germany) and analyzed using the FIJI distribution of the ImageJ software suite and the FigureJ plugin. The quantification of the tumor cells was performed on the principle of image segmentation by thresholding. For this, pictures were converted to a grayscale image and segmented into colored and uncolored pixels. If a pixel was above the threshold of luminosity, it was defined as white. If the luminosity value was below the threshold, which was true for all areas staining brown in the above immunohistochemistry, it was transformed into a red pixel. Areas with visibly necrotic features were excluded from the analysis.

### 4.6. Statistics

All experiments were run in biological and experimental replicates, with *n* ≥ 4 in each experiment. Statistical analyses were performed by Student’s t-test using SigmaPlot 10 (Systat, Chicago, IL, USA), with * = *p* < 0.05 and ** = *p* < 0.01.

## 5. Conclusions

We describe a facile and straightforward ex vivo system mimicking the in vivo situation of the tumor mass and the normal brain in GBM patients. It replaces animal studies and allows for the direct and reproducible application of test drugs and the precise quantitation of their effects on the bulk tumor mass as well as on the tumor’s invasive properties.

## Supplementary Materials

The following are available online at https://www.mdpi.com/2072-6694/12/9/2707/s1. Figure S1. Microscopic pictures of spheroid co-cultures and xenograft tissue slice tandem cultures (in triplicates) stained for Vimentin or other markers; Figure S2. Direct comparison of G55T2 xenograft tandem cultures grown on cortex vs. striatum; Figure S3. Microscopic pictures of spheroid co-cultures and xenograft tissue slice tandem-cultures (in triplicates) stained for Vimentin, based on different cell lines. Images were taken from tissue stained after one week of cultivation and show higher magnification of representative segments from Figure 1B,C.; Figure S4. Space occupying growth index, total tumor depth index (TTD-index) and space occupying growth depth index (SOGD-index) of spheroid co-cultures and xenograft tandem-cultures, based on different cell lines.; Figure S5. Vimentin staining of time-dependent tumor growth, invasion and proliferating cells in xenograft tandem-cultures.; Figure S6. Vimentin staining and quantitation of the tumor invasion index of tissue slice co-cultures based on spheroids from MZ-54 wildtype vs. STAT3 knockout (KO) cells. Reduction of tumor invasion index and space occupying growth index upon combined treatment of MZ-54 spheroid co-cultures with the inhibitors SGI-1776 (Pim1) and Stattic (STAT3).; Figure S7. Treatment of xenograft tandem-cultures based on U-87 MG or G55T2 cell xenografts with the Pim1 inhibitor SGI-1776 vs. solvent control. Representative microscopic pictures of Vimentin staining are shown (images from Figure 4 with dotted lines included for additional clarity, indicating the lower edge of the host tissue and the ‘horizon’ separating the upper and lower tumor area.
