# Supplementary material for: Glioblastoma Tissue Slice Tandem-Cultures for Quantitative Evaluation of Inhibitory Effects on Invasion and Growth
Source: Cancers (Basel). 2020 Sep 21;12(9):2707. doi: 10.3390/cancers12092707 (PMC7564455; doi:10.3390/cancers12092707)
Supplement: Supplementary file 1 [file cancers-12-02707-s001.pdf]

Supplementary Materials

Glioblastoma Tissue Slice Tandem-Cultures for Quantitative Evaluation of Inhibitory Effects on Invasion and Growth

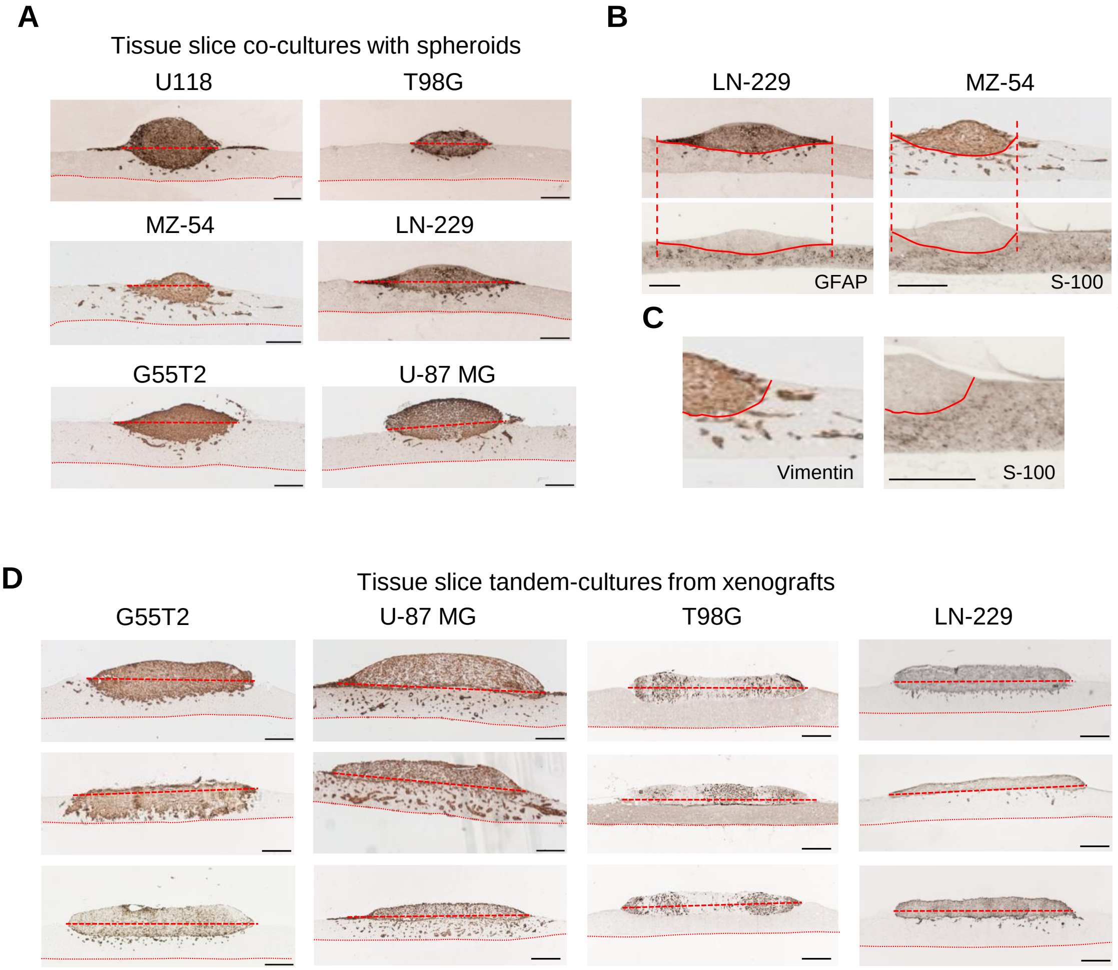

Figure S1. (**A–C**) Microscopic pictures of spheroid co-cultures and (**D**) xenograft tissue slice tandem-cultures (in triplicates) stained for Vimentin, based on different cell lines (images from Figure 1 with dotted lines included for additional clarity, indicating the lower edge of the host tissue and the 'horizon' separating the upper and lower tumor area). In (**B**), sequential sections stained for GFAP or S-100 are shown as well, and identical red lines in both corresponding sections indicating the border between tumor and underlying normal brain tissue are included for additional clarity. (**C**) The higher magnification, which again uses red lines for delineating the border between tumor and underlying normal brain tissue, shows infiltrating cells (right side of the left picture) which are clearly located in the normal brain tissue, as determined by the corresponding S-100 staining of normal tissue (right picture).

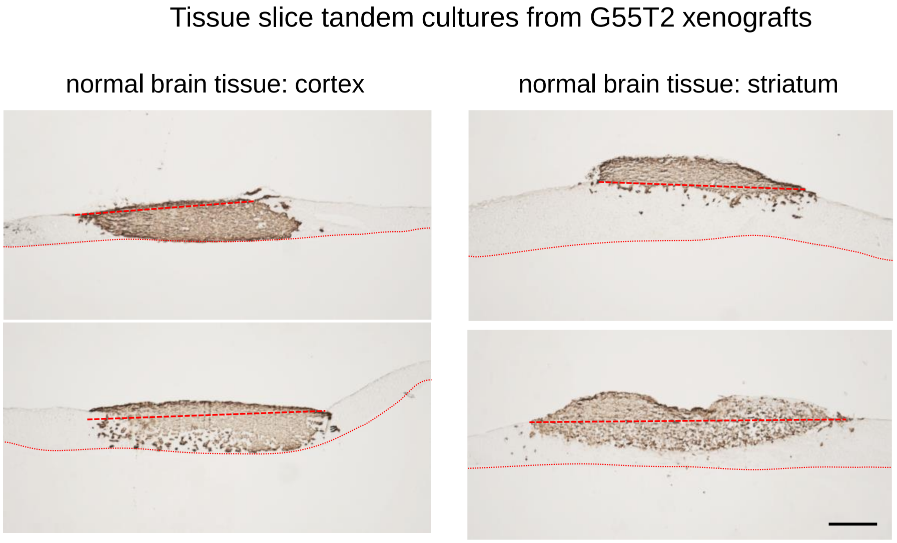

Figure S2. Direct comparison of G55T2 xenograft tandem-cultures grown on cortex (left) vs. striatum right) and stained for Vimentin. Scale bars: 200 µm.

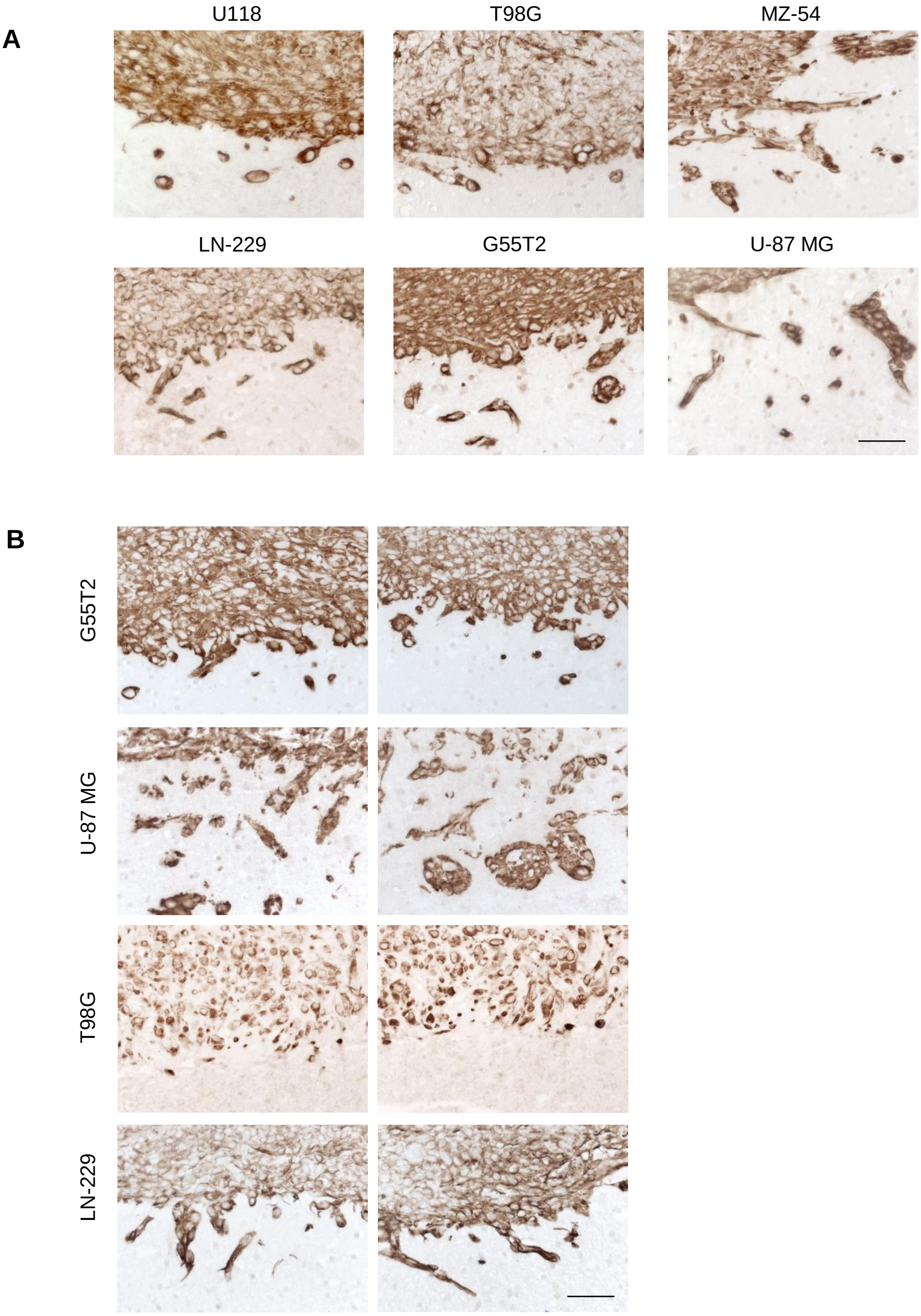

Figure S3. (**A**) Microscopic pictures of spheroid co-cultures and (**B**) xenograft tissue slice tandem-cultures (in triplicates) stained for Vimentin, based on different cell lines. Images were taken from tissue stained after one week of cultivation and show higher magnification of representative segments from Figure 1B,C. Scale bar: 50 µm.

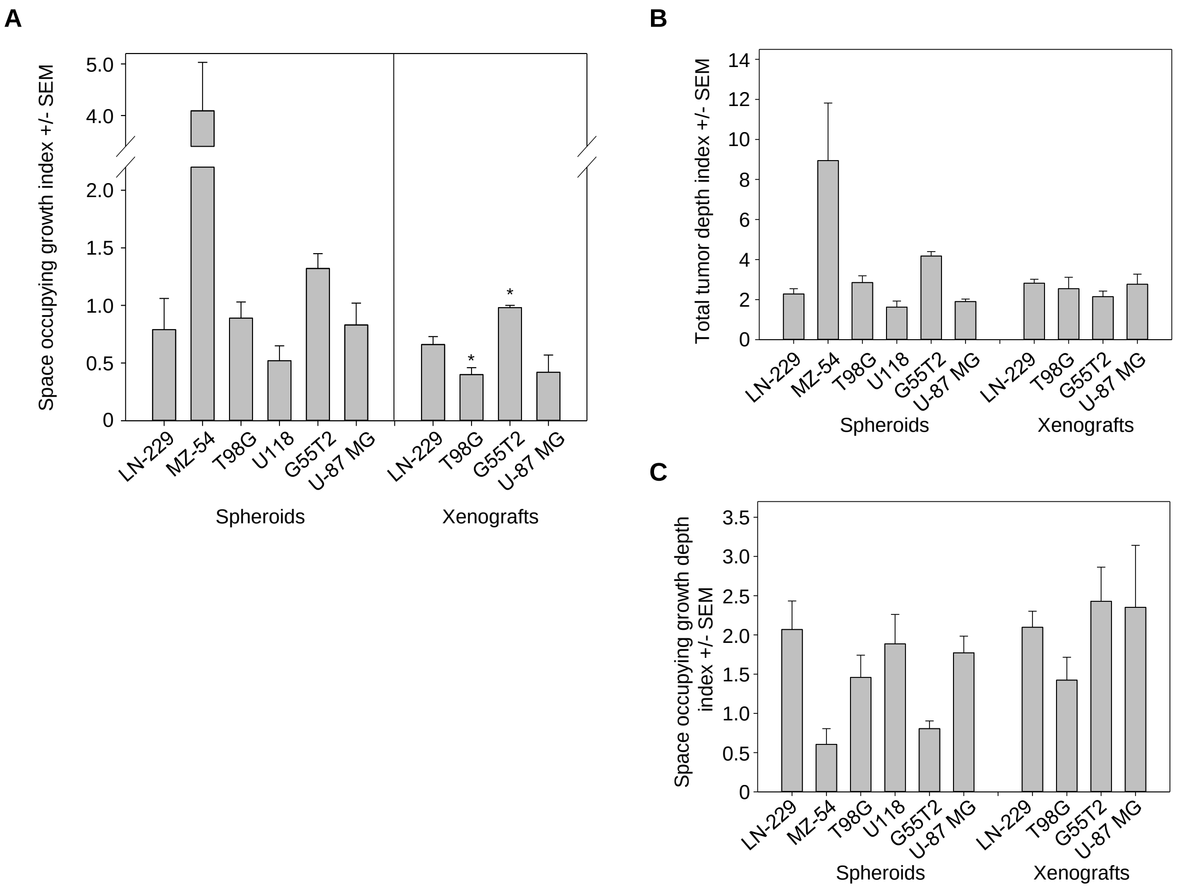

Figure S4. (**A**) Space occupying growth index of spheroid co-cultures and xenograft tandem-cultures, based on different cell lines. Symbols indicate statistically significant (*) differences of xenograft tandem-cultures vs. their spheroid co-culture counterparts. (**B**) Total tumor depth index (TTD-index); (**C**) Space occupying growth depth index (SOGD-index).

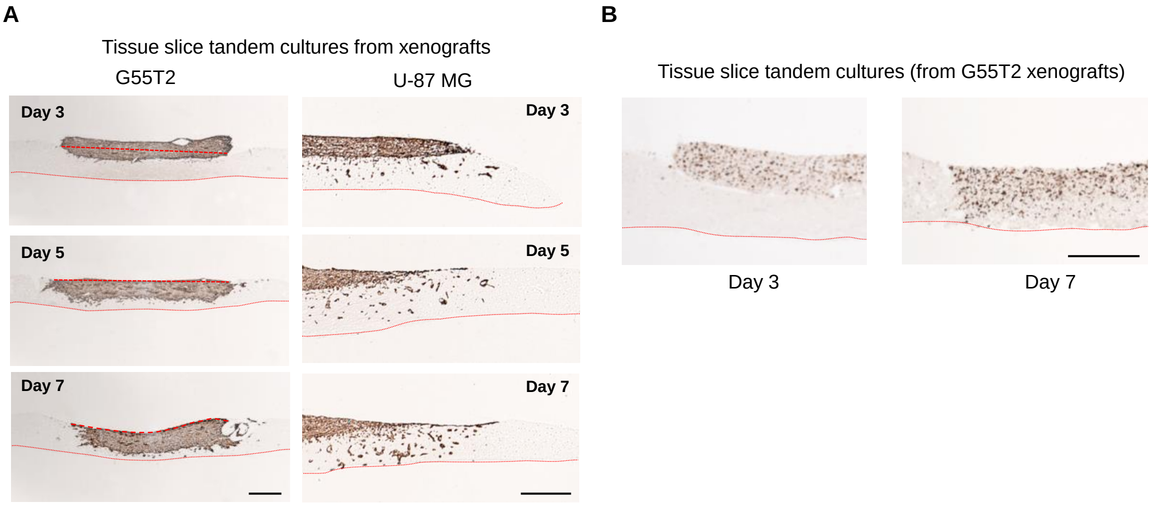

Figure S5. (**A**) Vimentin staining of time-dependent tumor growth and invasion in xenograft tandem-cultures, based on G55T2 (left) or U-87 MG cells (right). (**B**) Proliferating cells in G55T2 xenograft tandem-cultures, as determined by Ki-67 staining. Scale bars: 200 µm (images from Figure 3 with dotted lines included for additional clarity, indicating the lower edge of the host tissue and the 'horizon' separating the upper and lower tumor area).

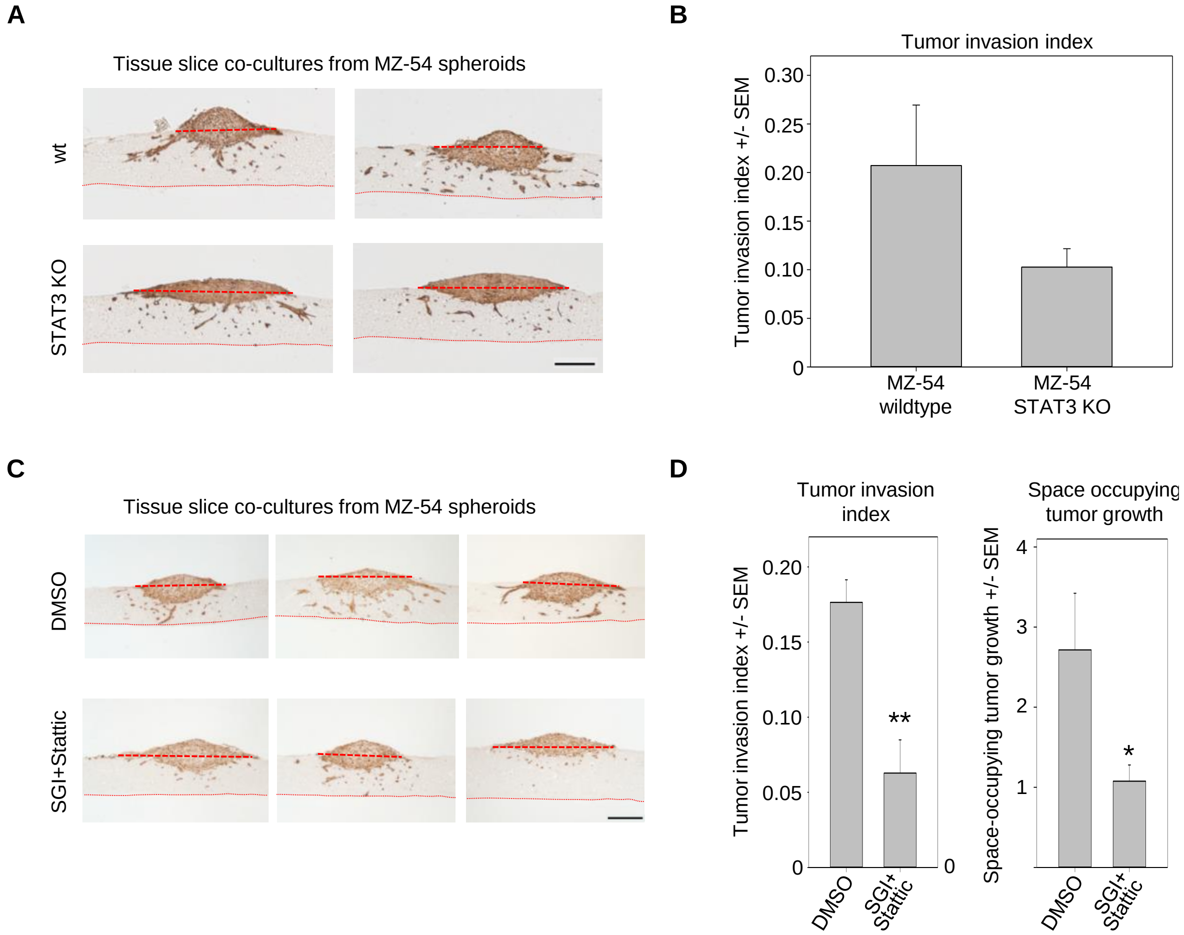

Figure S6. Vimentin staining (**A**) and quantitation of the tumor invasion index (**B)** of tissue slice co-cultures based on spheroids from MZ-54 wildtype (WT) vs. STAT3 knockout (KO) cells. (**C**,**D**) Reduction of tumor invasion index and space occupying growth index upon combined treatment of MZ-54 spheroid co-cultures with the inhibitors SGI-1776 (Pim1) and Stattic (STAT3). (**C**): microscopic pictures stained for Vimentin; (**D**): quantitation of tumor invasion index and space occupying tumor growth. Dotted lines indicating the lower edge of the host tissue and the 'horizon' separating the upper and lower tumor area have been included for additional clarity; scale bars: 200 µm.

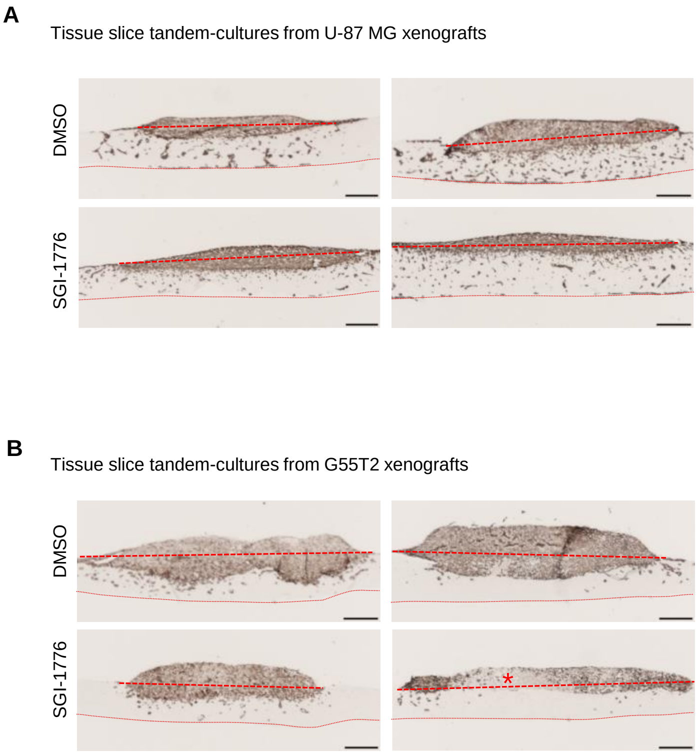

Figure S7. Treatment of xenograft tandem-cultures based on (**A**) U-87 MG or (**B**) G55T2 cell xenografts with the Pim1 inhibitor SGI-1776 vs. solvent control. Representative microscopic pictures of Vimentin staining (asterisk: necrotic area) are shown (images from Figure 4 with dotted lines included for additional clarity, indicating the lower edge of the host tissue and the 'horizon' separating the upper and lower tumor area. Scale bars: 200 μm.
